# Supplementary material for: A 16th century Escherichia coli draft genome associated with an opportunistic bile infection
Source: Commun Biol. 2022 Jun 16;5:599. doi: 10.1038/s42003-022-03527-1 (PMC9203756; doi:10.1038/s42003-022-03527-1)
Supplement: Supplementary file 2 — Supplementary Information [file 42003_2022_3527_MOESM2_ESM.pdf]

Supplementary Appendix to: A 16th Century *Escherichia coli* draft genome associated with an opportunistic bile infection

George S. Long[1,2,*,†], Jennifer Klunk[1,2,3,*], Ana T. Duggan[2,4], Madeline Tapson[2,3], Valentina Giuffra[5], Lavinia Gazzè[6], Antonio Fornaciari[7], Sebastian Duchene[8], Gino Fornaciari[7], Olivier Clermont[9], Erick Denamur[9,10,†], G. Brian Golding[1], Hendrik Poinar[2,11,12,†]

May 15, 2022

[1] Department of Biology, McMaster University, Hamilton, Canada
[2] McMaster Ancient DNA Centre, Departments of Anthropology and Biochemistry, McMaster University, Hamilton, Canada
[3] Daicel Arbor Biosciences, 5840 Interface Drive, Suite 101, Ann Arbor, MI 48103, USA
[4] Department of Anthropology, McMaster University, Hamilton, Canada
[5] Division of Paleopathology, Department of Translational Research and New Technologies in Medicine and Surgery, University of Pisa, Via Roma 57, 56126 Pisa, Italy
[6] Department of Human Science (DISUM), University of Catania, Piazza Dante 32, 95124, Catania, Italy
[7] Department of Civilisations and Forms of Knowledge, University of Pisa, Via Trieste 40, 56126, Pisa, Italy
[8] Department of Microbiology and Immunology, Peter Doherty Institute for Infection and Immunity, University of Melbourne, Melbourne, Australia
[9] Université de Paris, IAME, UMR 1137, INSERM, 75018 Paris, France
[10] Laboratoire de Génétique Moléculaire, Hôpital Bichat, APHP, 75018 Paris, France
[11] Michael G. DeGroote Institute for Infectious Disease Research and CIFAR Humans and the Microbiome Program, Toronto, Canada
[12] CIFAR Humans and the Microbiome Program, Toronto, Canada
[*] These authors contributed equally to this work
[†] Corresponding Authors: George S. Long (longg2@mcmaster.ca), Erick Denamur (erick.denamur@inserm.fr), and Hendrik Poinar (poinarh@mcmaster.ca)

Contents

Supplementary Notes **3**

Supplementary Methods **3**

Supplementary Discussion **5**

Supplementary References **6**

Supplementary Tables **7**

Supplementary Figures **8**

Supplementary Notes

Giovanni d'Avalos, born in 1538, was the fourth son of Alfonso d'Avalos (1502-1546) and Maria d'Aragona (1503-1568). His father Alfonso d'Avalos, Marquis del Vasto, was governor of Milan (1538-1546) and a famous commander in the army of Emperor Charles V. Originally raised in Milan, Giovanni lived with his family in Naples and Ischia following the death of his father. In 1565, he married the noblewoman Maria Orsini, daughter of the Duke of Gravina in Matera (southern Italy)[1]. Giovanni left Naples in 1568, moving to Palermo to follow elder brother Francesco Ferdinando who became viceroy of Sicily, where he remained until 1571[2,3].

After the naval battle of Lepanto, against the Turks, in 1572 he mentioned in Sicily in the retinue of Don Juan d'Austria to plan the conquest of Tunis[2]. It is likely that, starting from 1574, he began to have health problems, because during the stay of Don Juan d'Austria in Naples (1574-76) he did not participate in tournaments or court life, where the brothers Cesare and Carlo d 'Avalos are present instead[4].

Giovanni had no children and, perhaps due to his illness, devoted himself to religious works in the last period of his life. In 1585, he founded the hermitage of SS. Salvatore on the hill of Camaldoli in Naples. He died in 1586. Stable isotopes of carbon and nitrogen in bone collagen reveal a diet particularly rich in meat[5], with a low intake of vegetables and with an integration of sea fish of about 16%[6].

The paleopathological study shows: obesity, severe pulmonary anthracosis, fatty infiltration of the myocardium and myocardial fibrosis, probably from ischemic disease, chronic arterial disease, with atheromas of the thoracic aorta, mixed stones of the gallbladder, with chronic cholecystitis and liver fibrosis. The wall of the calculus gallbladder is thickened and completely de-epithelialized, with the presence of intraparietal Rokitansky-Aschoff sinuses[7]. These are tubular structures representing herniations or diverticula which result from increased intraluminal pressure[8,9].

Supplementary Methods

The samples taken from NASD1 consisted of several gallstones, one larger than the others, of a dark brown, almost black, color, with an appearance similar to coal. The entirety of the largest stone (87.4 mg) was taken whole through demineralization (DEM) and digestion (DIG) without additional pulverization or crushing so that each subsequent demineralization and digestion round would represent progressively more interior layers of the nodule. The selected gallstone from NASD1 was processed alongside seven other nodules (not included in this publication) and a single reagent blank was carried through all downstream processes for the eight nodules.

The nodule was demineralized by adding 0.5 mL of EDTA (0.5 M, pH 8) to the sample and then incubating for 24 hours at 22°C with agitation at 1000 rpm. The samples were centrifuged at 13,200 rpm for 3 minutes, at which time the supernatant was collected and stored at -20°C. A volume of 0.5 mL of digestion buffer (20 mM Tris-HCl pH 8.0, 0.5% sarcosyl, 250 μg/mL Proteinase K, 5 mM $CaCl_2$, 5 0mM dithiothreitol (DTT), 1% polyvinylpyrrolidone (PVP), 2.5 mM N-phenacylthiazolium bromide (PTB)[10] was added to the remaining sample material, which was then incubated for 24 hours at 25°C with agitation at 1000 rpm. The supernatant collection was performed again. A total of six rounds each of DEM and DIG were performed with DEM and DIG supernatants stored separately. Visually, the supernatant for the first two rounds of DEM and DIG were extremely dark in color and slightly viscous. The first round of DEM and DIG contained a small amount of solids. Later DEM and DIG supernatants became increasingly clearer in colour and less viscous, though there was a persistent faint yellow tinge to the coloration.

Extraction was performed following a protocol designed to retain short molecules[11] with the modification of using a High Pure Viral Nucleic Acid Large Volume column (Roche) as suggested by Glocke & Meyer 2017 instead of a modified Minelute column. An aliquot of 250 μL was taken from each of the third and fourth DEM and DIG supernatants for a total volume of 1 mL, which was combined with 13 mL of a guanidinium-hydrochloride buffer (5 M guanidine hydrochloride, 40% (vol/vol) isopropanol, 0.05% Tween-20, and 90 mM sodium acetate (pH 5.2). This mixture was spun through the column for 4 minutes at 1,500 x g. The flow-through was discarded, then the other half of the volume was added to the column and spun again for 4 minutes at 1,500 x g. The columns were rotated 90° and spun for an additional 2 minutes at 1,500 x g. The apparatus was disassembled and the column portion placed in a clean 2-mL collection tube. The columns were dry spun for 1 minute at 6,000 rpm. 650 μL of Qiagen Buffer PE was added to the column, which was then spun for 1 minute at 6,000 rpm. The flow-through was discarded, and then the wash with Buffer PE was repeated. The column was dry spun for 30 seconds at maximum speed (13,200 rpm), rotated 180°, and dry spun for an additional 30 seconds at maximum speed. The column was transferred to a clean 1.75-mL tube, then 20 μL of Qiagen Buffer EB was added to the center of the membrane and incubated for 5 minutes at room temperature. The column was spun for 1 minute at maximum speed. The elution step was repeated a second time for a total of 40 μL of extract.

Library preparation and double-indexing were performed according to Meyer & Kircher 2010 and Kircher et al. 2011 with several modifications. Reactions were performed in 40 μL volumes. 10 μL of extract was used as template into blunt end repair. Purification after blunt end repair was performed with the QiaQuick Nucleotide Removal kit (Qiagen) with an elution volume of 20 μL. Adapter ligation was performed with a final concentration of 0.25 μM adapters and the reaction was run overnight for 15 hours. Purification after adapter ligation was performed using a Minelute PCR Purification kit (Qiagen) with an elution volume of 20 μL. The fill-in step was deactivated by heat (80°C for 20 minutes) and used directly as input into the indexing reaction. An additional reagent blank was incorporated at the beginning of library preparation.

All purifications using kits from Qiagen during library preparation and in all downstream steps included the following modifications: binding and washing spins were performed at 6,000 rpm, 650 μL of Buffer PE was used for washing and the washing step was performed twice, dry spins were performed for 30 seconds at maximum speed (13,200 rpm), rotated 180°, and dry spun for an additional 30 seconds at maximum speed, before elution, the buffer was allowed to incubate on the membrane for 5 minutes at room temperature.

Dual-7bp indexing was performed in 40 μL reactions using 20 μL of KAPA SYBR® FAST qPCR Master Mix (2X), 3 μL each of 10 μM forward and reverse indexes (final concentration: 750 nM), 4 μL of water, and 10 μL of template. Following a 5 minute initialization at 95°C, 12 cycles of 95°C for 30 seconds and 60°C for 45 seconds was run. After a final extension at 60°C for 3 minutes, samples were cooled to 4°C before purification with a Minelute PCR Purification Kit (Qiagen) using the aforementioned modifications and an elution volume of 15 μL.

The libraries were quantified post-indexing in 10 μL reactions consisting of 5 μL of KAPA SYBR® FAST qPCR Master Mix (2X), 0.2 μL each of 10 μM forward and reverse primers (IS5_long_amp.P5 and IS6_long_amp.P7 from Meyer & Kircher 2010), 0.6 μL of water, and 4 μL of template (indexed library diluted 1 in 10,000 with EBT). The standard for this assay was PhiX (Illumina) serially diluted to 100 pM, 10 pM, 1 pM, 500 fM, 250 fM, 125 fM, and 62.5 fM. The cycling conditions were the same as the indexing conditions, except 35 cycles were run instead of 12.

The indexed library from NASD1 and the associated blanks were pooled at roughly equimolar ratios with libraries from other projects (including the other nodule libraries), diluting samples in EB if the required volume was less than 0.5 μL. The pool was concentrated using a Minelute PCR Purification kit (Qiagen) and eluted in 12 μL of buffer EB. Size selection was performed using a 3% NuSieve GTG gel run for 35 minutes at 100V. The band between 150-500bp was excised and purified using a Minelute Gel Extraction kit (Qiagen) and eluted in 20 μL of buffer EB.

The sequencing pool was delivered to the Farncombe Metagenomics Facility (McMaster University), where it was assessed for concentration and quality and then sequenced on a partial run on the Illumina HiSeq 1500 platform with paired-end 90 bp reads. The reads were delivered in demultiplexed format using Illumina's bcl2fastq software.

After the initial data processing indicated the potential presence of *E. coli*, more reads were needed to confirm the presence and allow for deeper analysis. An additional aliquot of the original library (produced from DEM/DIG rounds three and four), exhausting the rest of the remaining volume, was pooled with other libraries, size-selected, and sequenced again as above. The overall sequencing depth of the gallstone was $61,456,824$ reads (see supplemental table 1 for the breakdown of reads by digest).

To determine whether DEM and DIG rounds prior to or subsequent to the rounds sequenced (three and four) contained a larger or smaller proportion *E. coli* DNA, a second set of samples was processed. The following volumes of DEM and DIG supernatants were taken through the same extraction procedure as above: round 1 – 250 μL each of DEM and DIG for a total of 500 μL, round 2 – 250 μL each of DEM and DIG for a total of 500 μL, rounds 5 and 6 (combined) – 250 μL each of DEM and DIG for a total of 1 mL. Library preparation, indexing, size selection, and sequencing were performed again as above. During indexing, a second aliquot of unindexed library from the third and fourth rounds of DEM and DIG was included in the workflow.

Three additional tissue types from NASD1 were processed: NASD1 1/14, 32.5 mg of urinary/bladder tissue, NASD1 1/15/B, 27.3 mg of small intestine, NASD1 1/11/A, 75.2 mg of lung tissue. The urinary tissue was reddish, clung to the container, and retained the odour of urine. The small intestine tissue was tan-coloured and brittle. The lung tissue was medium brown in color and very brittle with some potentially calcified sections. These tissues were cut into smaller pieces with a clean scalpel and digested for four rounds. All 2 mL of supernatant was combined for extraction, which was then processed through sequencing as above. During the indexing for these alternate tissue types, a third (and final) aliquot of unindexed library from the third and fourth rounds of DEM/DIG of the nodule was incorporated, though it was sequenced separately.

To increase the material available for sequencing, a second library was prepared from another 10 μL of extract from DEM/DIG rounds 3 and 4. Two 10 μL aliquots of this library were indexed with different combinations as above and pooled for size selection and sequencing along with the third index combination from the first library from DEM/DIG rounds 3 and 4.

A final round of size-selection and sequencing was performed on additional aliquots of the libraries from DEM/DIG rounds 1, 2, 5/6, as well as four libraries from 3/4 (two from each preparation).

Supplementary Discussion

Sequence typing of our ancient *E. coli* strain was done using the Center for Genomic Epidemiology (CGE) MLST service[12]. Both MLST schemes – Warwick University and Pasteur Institute – were tested using the mapped global pan-genome reads. Only an incomplete typing was achieved due to indels, however, the information obtained confirmed the placement of our ancient strain in the A0 subroup phylogeny. The Warwick scheme indicated that our strain is likely a member of ST4995 (see Supplemental table 2 for the MLST), the same sequence type as its sister taxa. The Pasteur Institute results potentially correspond to ST1022 (see Supplemental table 3 for the full MLST). A *fimH* variant – *fimH*86 – was determined using the CGE as well[13], indicating a different variant from its sister strains. The serotype was identified as per the method outlined in Ingle *et al.* 2016, returning the serotype Onovel15:H?[14]. This serotype is also similar (Onovel15:H16) to that found in the clade as our ancient strain.

Supplemental References

1 Volpe, F. *Memorie storiche profane religiose su la città di Matera*, 171 (Stamperia Simonia, 1818).

2 Paruta, F. & Palmerino, N. *Diario della città di Palermo (1550-1613)*, vol. 1, 30, 48 (1869).

3 Mori, E. & L'Archivo Orsini. *La Famiglia, la Storia, l'Inventario* (Viella, 2016).

4 Vaini, E. Reports to Francesco I de' Medici in Correspondence (1573-1574).

5 Fornaciari, G. Food and disease at the Renaissance courts of Naples and Florence: a paleonutritional study. *Appetite* **51**, 10–14; 10.1016/j.appet.2008.02.010 (2008).

6 Richards, M. P. & Hedges, R. E. Stable isotope evidence for similarities in the types of marine foods used by Late Mesolithic humans at sites along the Atlantic coast of Europe. *Journal of Archaeological Science* **26**, 717–722; 10.1038/nmeth.4285 (1999).

7 Fornaciari, G., Pollina, L., Tornaboni, D. & Tognetti, A. Pulmonary and hepatic pathologies in the series of mummies of S. Domenico Maggiore at Naples (XVI century). In *Proceedings of the VII European Meeting of the Paleopathology Association (Lyon, September 1988)*, vol. 1, 89–92. Marino Solfanelli Editore (Journal of Paleopathology, Monographic Publications, 1989).

8 Rosai, J. Gallbladder and extrahepatic bile ducts. In *Ackerman's Surgical Pathology*, vol. 1, 949 (Mosby, St. Louis, Missouri, 1996), 8 edn.

9 Robertson, H. & Ferguson, W. J. The diverticula (Luschka's crypts) of the gallbladder. *Archives of Pathology* **40**, 312–333 (1945).

10 Schwarz, C. *et al.* New insights from old bones: DNA preservation and degradation in permafrost preserved mammoth remains. *Nucleic Acids Research* **37**, 3215–3229; 10.1093/nar/gkp159 (2009).

11 Dabney, J. *et al.* Complete mitochondrial genome sequence of a Middle Pleistocene cave bear reconstructed from ultrashort DNA fragments. *Proceedings of the National Academy of Sciences* 201314445; 10.1073/pnas.1314445110 (2013).

12 Larsen, M. V. *et al.* Multilocus sequence typing of total-genome-sequenced bacteria. *Journal of Clinical Microbiology* **50**, 1355–1361; 10.1128/JCM.06094-11 (2012).

13 Roer, L. *et al.* Development of a web tool for *Escherichia coli* subtyping based on fimH alleles. *Journal of Clinical Microbiology* **55**, 2538–2543; 10.1128/JCM.00737-17 (2017).

14 Ingle, D. J. *et al.* In silico serotyping of *E. coli* from short read data identifies limited novel O-loci but extensive diversity of O: H serotype combinations within and between pathogenic lineages. *Microbial Genomics* **2**; 10.1099/mgen.0.000064 (2016).

15 Wirth, T. *et al.* Sex and virulence in *Escherichia coli*: an evolutionary perspective. *Molecular Microbiology* **60**, 1136–1151; 10.1111/j.1365-2958.2006.05172.x (2006).

16 Jónsson, H., Ginolhac, A., Schubert, M., Johnson, P. L. & Orlando, L. mapDamage2.0: fast approximate Bayesian estimates of ancient DNA damage parameters. *Bioinformatics* **29**, 1682–1684; doi:10.1093/bioinformatics/btt193 (2013).

Supplementary Tables

Supplemental Table 1: Pooled raw sequencing depths of the gallstone libraries.

Sample	Reads
Digest 1	8,027,378
Digest 2	7,418,026
Digests 3 & 4	39,577,746
Digests 5 & 6	6,433,674
Blank Digests 3 & 4	1,209,503
Blank Digests 5 & 6	564,487
Library Blank	571,512

Supplemental Table 2: Sequence type of the ancient *E. coli* strain based on the Warwick University scheme[15].

ST	*adk*	*fumC*	*gyrB*	*icd*	*mdh*	*purA*	*recA*
ST4995-like	6	4	5	1_{14}	8	8	6

Subscript values indicate the number of missing nucleotides in a locus.

Supplemental Table 3: Sequence type of the ancient *E. coli* strain based on the Institut Pasteur Scheme.

ST	*dinB*	*icd*	*pabB*	*polB*	*putP*	*trpA*	*trpB*	*uidA*
ST1022-like	8_1	323	110	17_1	16	1	201	40

Subscript values indicate the number of missing nucleotides in a locus.

Supplementary Figures

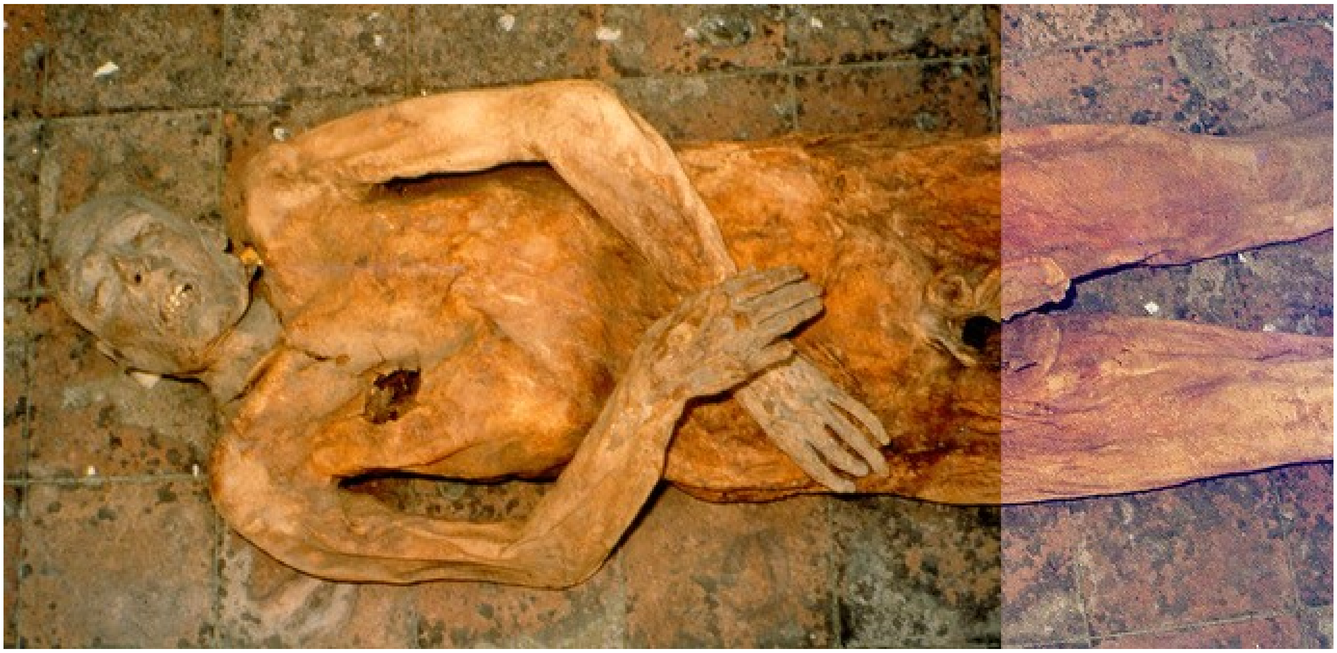

Supplemental Figure 1: **External examination of Giovanni d'Avalos (NASD1)**

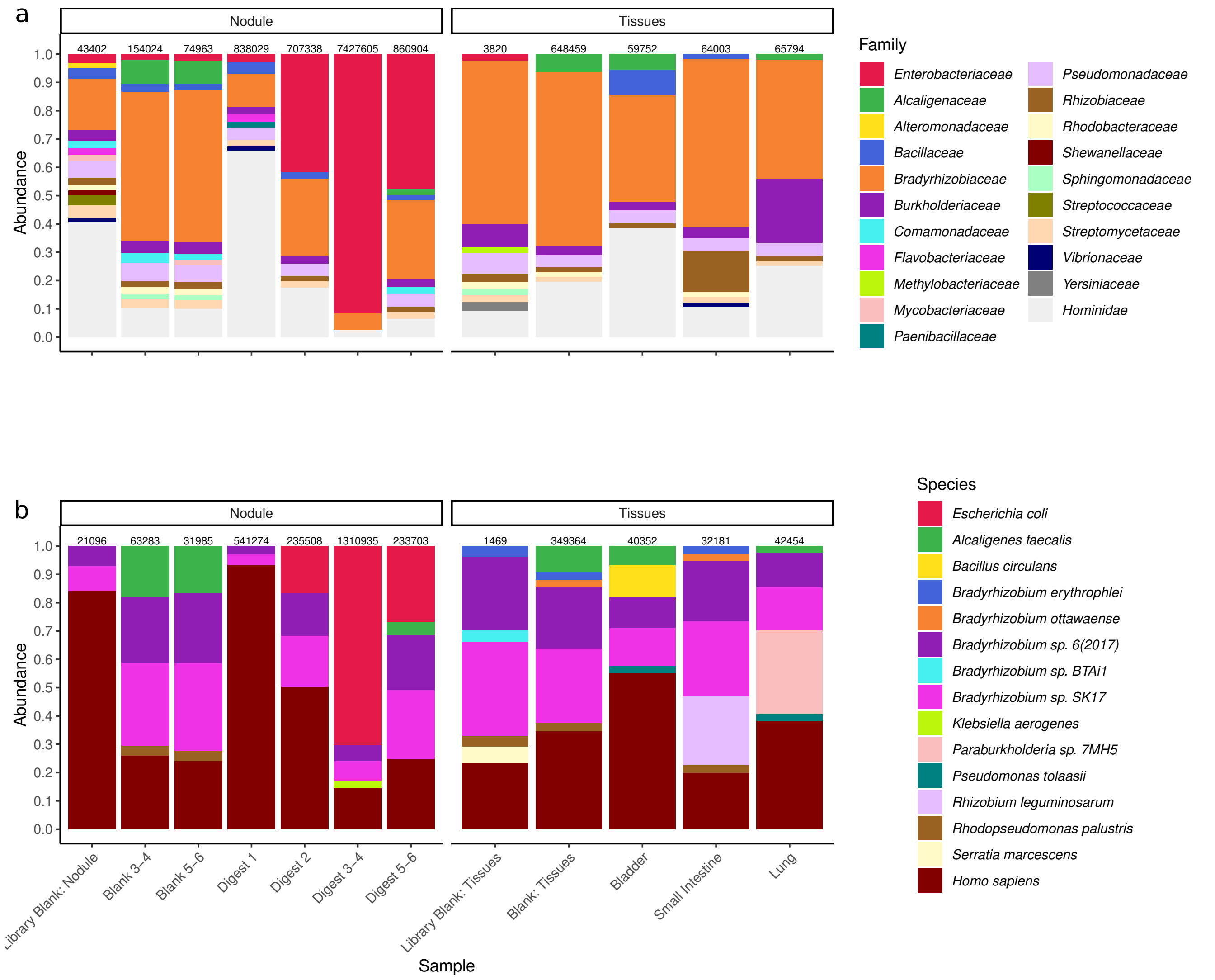

Supplemental Figure 2: **Proportional taxon abundances of NASD1 samples per** `Kraken2`. **a)** represents counts at the family level while **b)** is for the species level. A 1% – 1.2% when plotting the family results – abundance filter was used to filter out low-abundance taxa. The numbers at the top of each sample indicates the count of reads which were classified at either taxonomic level. Unclassified reads were ignored when plotting. See Supplemental Figure 14 for the results with unclassified reads, the low abundance taxa, and context into the total proportion of less specific reads.

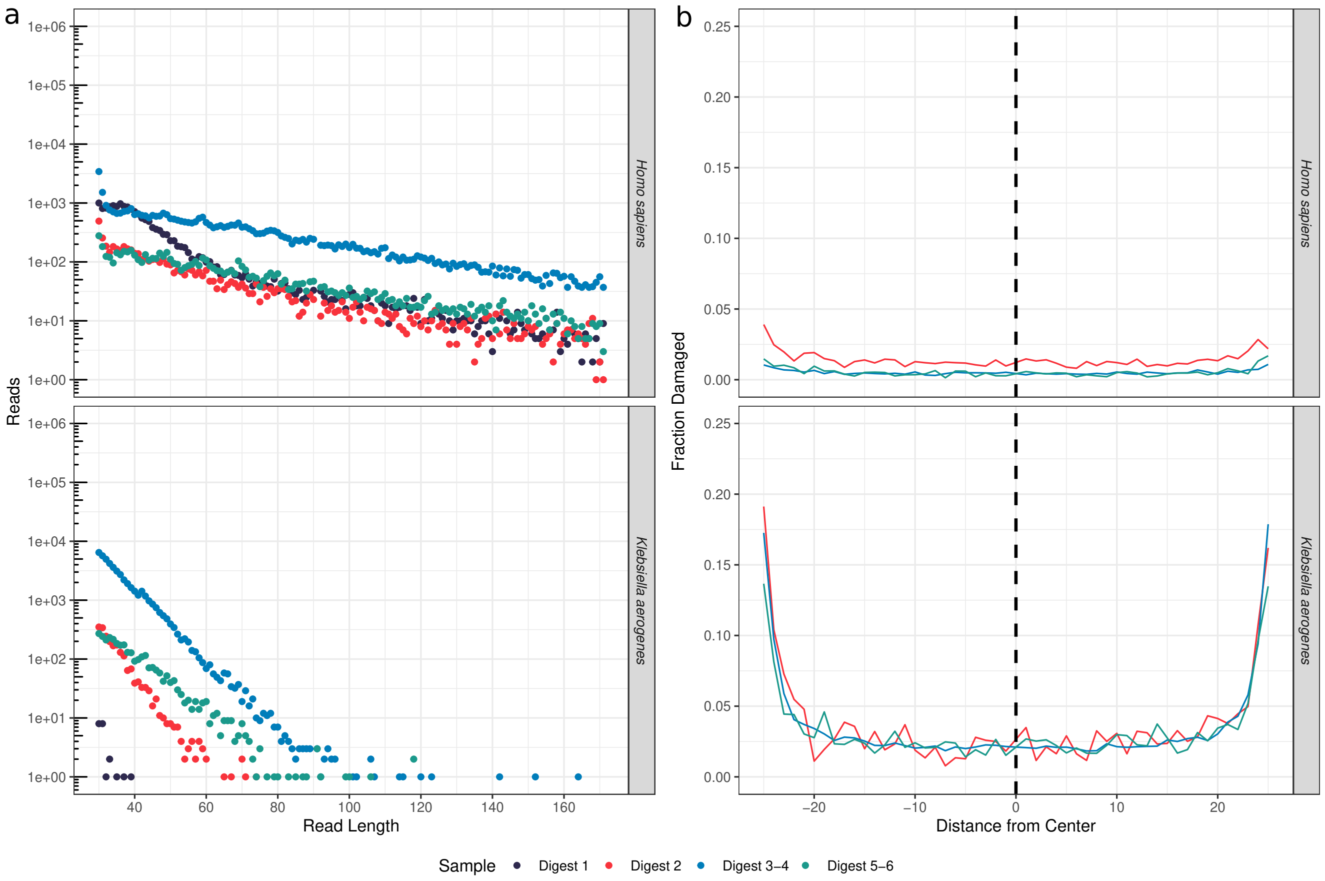

Supplemental Figure 3: **Additional Ancient DNA Authentication**. **a)** Fragment length distribution of deduplicated mapped reads from *H. sapiens* and *K. aerogenes*. A $\log_{10}$ scale is used to emphasize the differences between the digests. A minimum length of 30bp was required for a read to be kept. **b)** Damage plots of the 5′ and 3′ ends of mapped reads for *E. coli*. Colours refer to the digest, **not** damage type. `Mapdamage 2.0`[16] was used to calculate the damage rates.

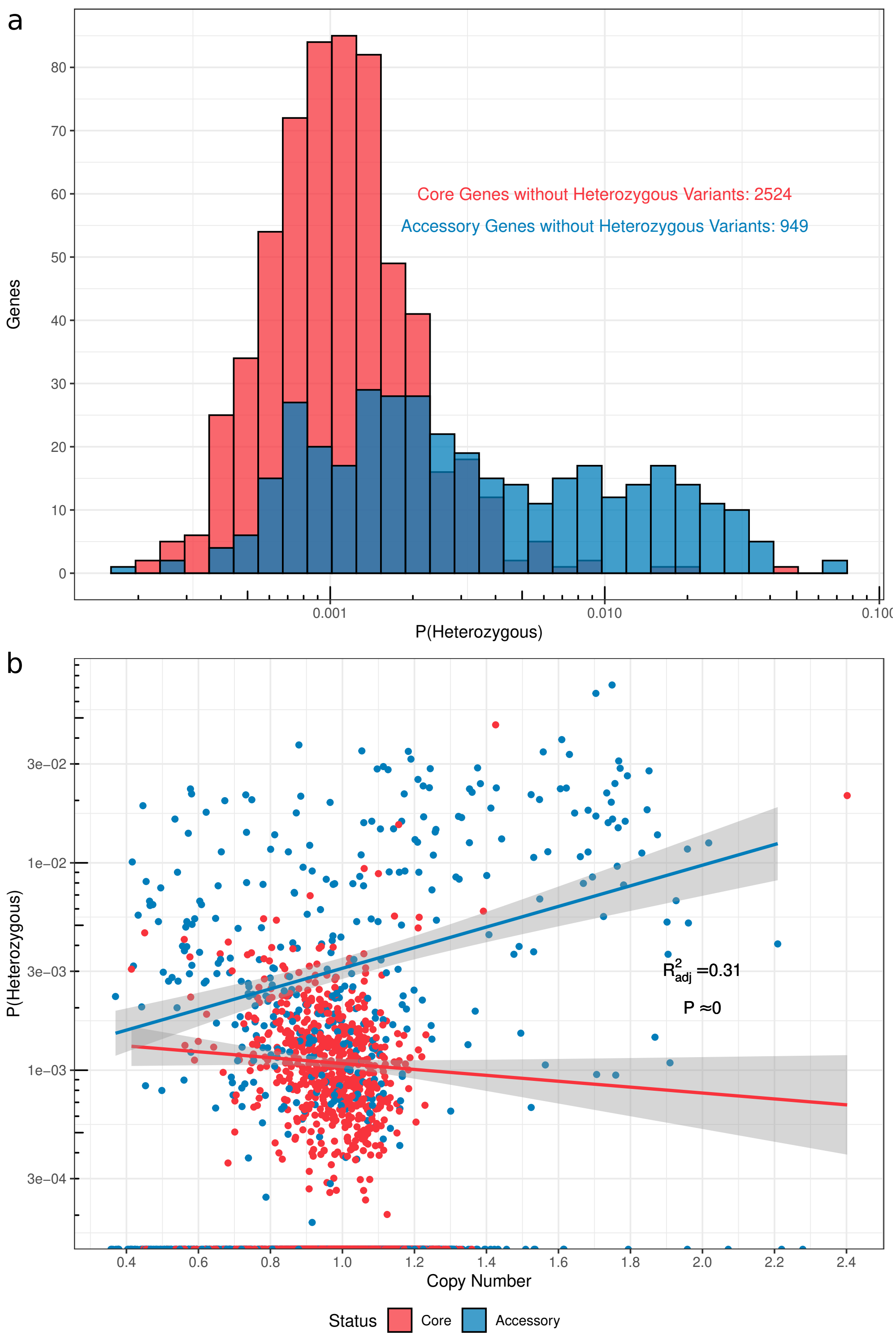

Supplemental Figure 4: **Gene heterozygosity metrics for the core and accessory genomes.** **a)** Histogram of gene heterozygosity for the core and accessory genomes. Genes without heterozygous SNPs were excluded from the histogram. **b)** A scatter plot demonstrating the relationship of gene copy numbers and heterozygosity. The R^2_{adj} and P-value were calculated from the following linear model: $\log_{10}(F(Heterozygosity)) \sim CopyNumber : Core$ where Core is a categorical variable and genes without heterozygosity were excluded. The shaded regions represent the 95% confidence interval.

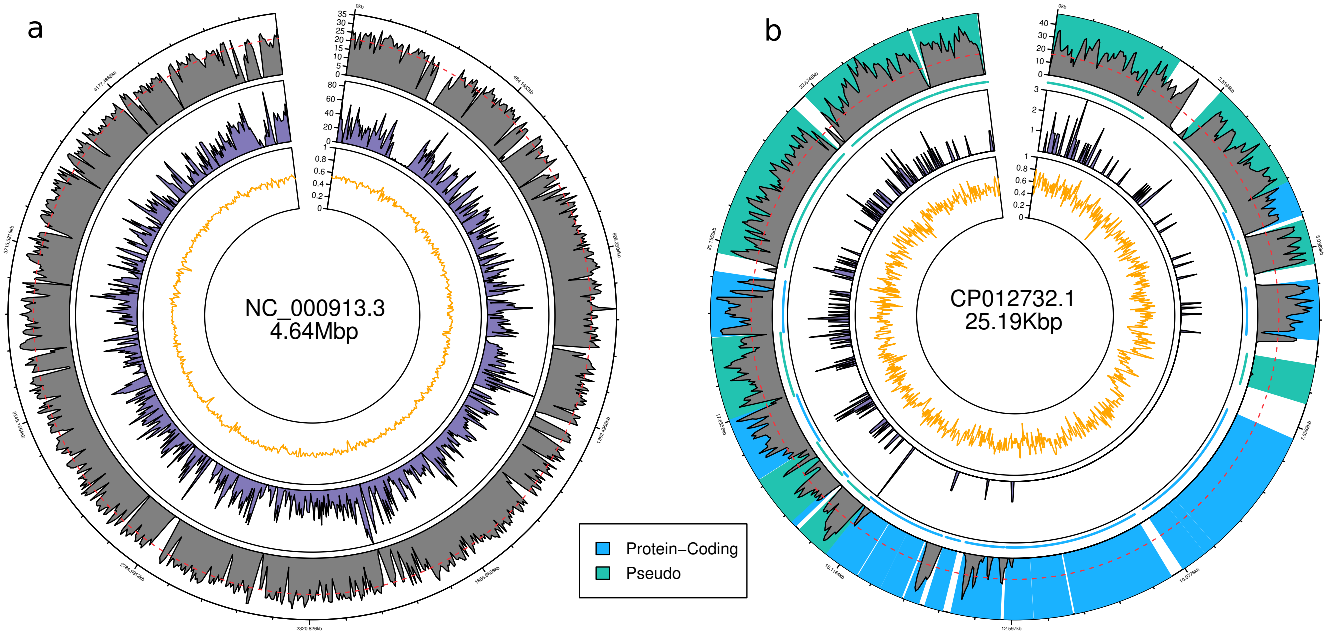

Supplemental Figure 5: **Additional Sequence Coverage Plots**. Rolling mean coverages of *E. coli* K-12 MG1655 (**a**) and the `CP012732.1` plasmid (**b**). The first track indicates the coverage with the red line illustrating the overall mean. The second track indicates the number SNPs over the same window while the third is the GC content. A window of 0.1% was used for illustrative purposes.

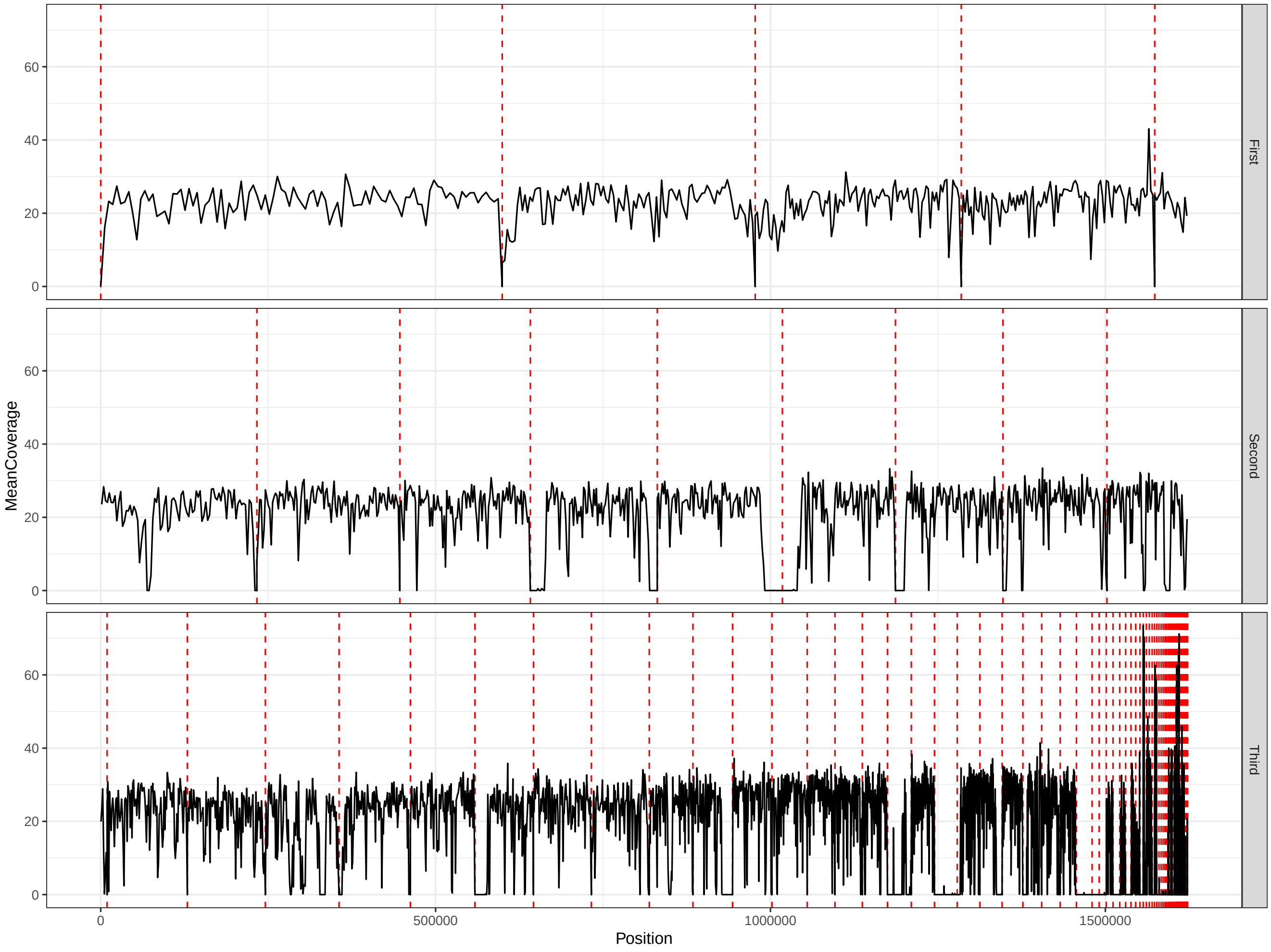

Supplemental Figure 6: **Mean Coverage of FSIS11816402 Scaffolds**. Mapping depths of the `FSIS11816402` contigs. A 1% mean was used for each contig. Each plot represents a third of the total assembly length. The red dashed line indicates the start of a new contig.

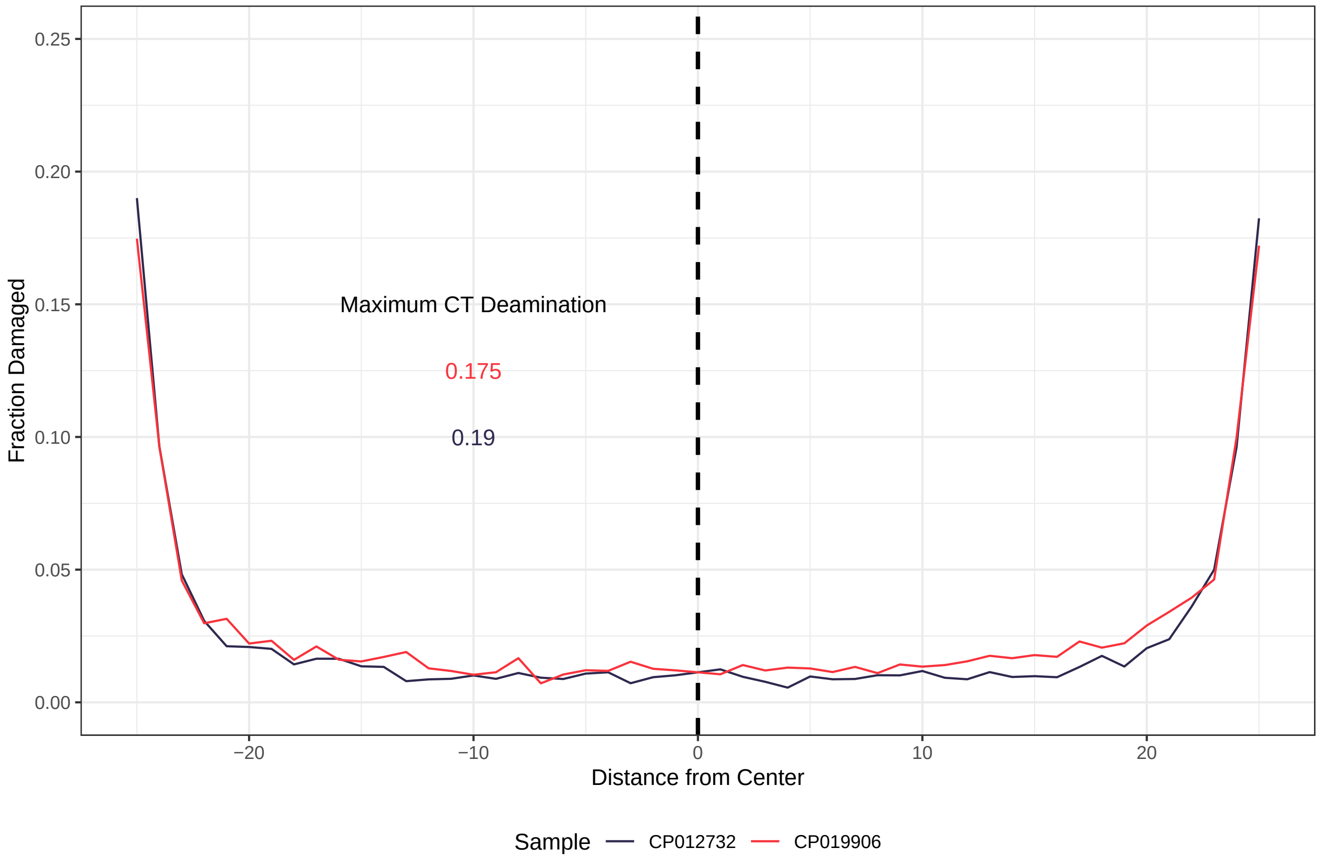

Supplemental Figure 7: **Plasmid Ancient DNA Authentication**. Damage plots of the 5′ and 3′ ends of mapped reads for `CP012732` and `CP019906`. Colours refer to the digest, **not** damage type.

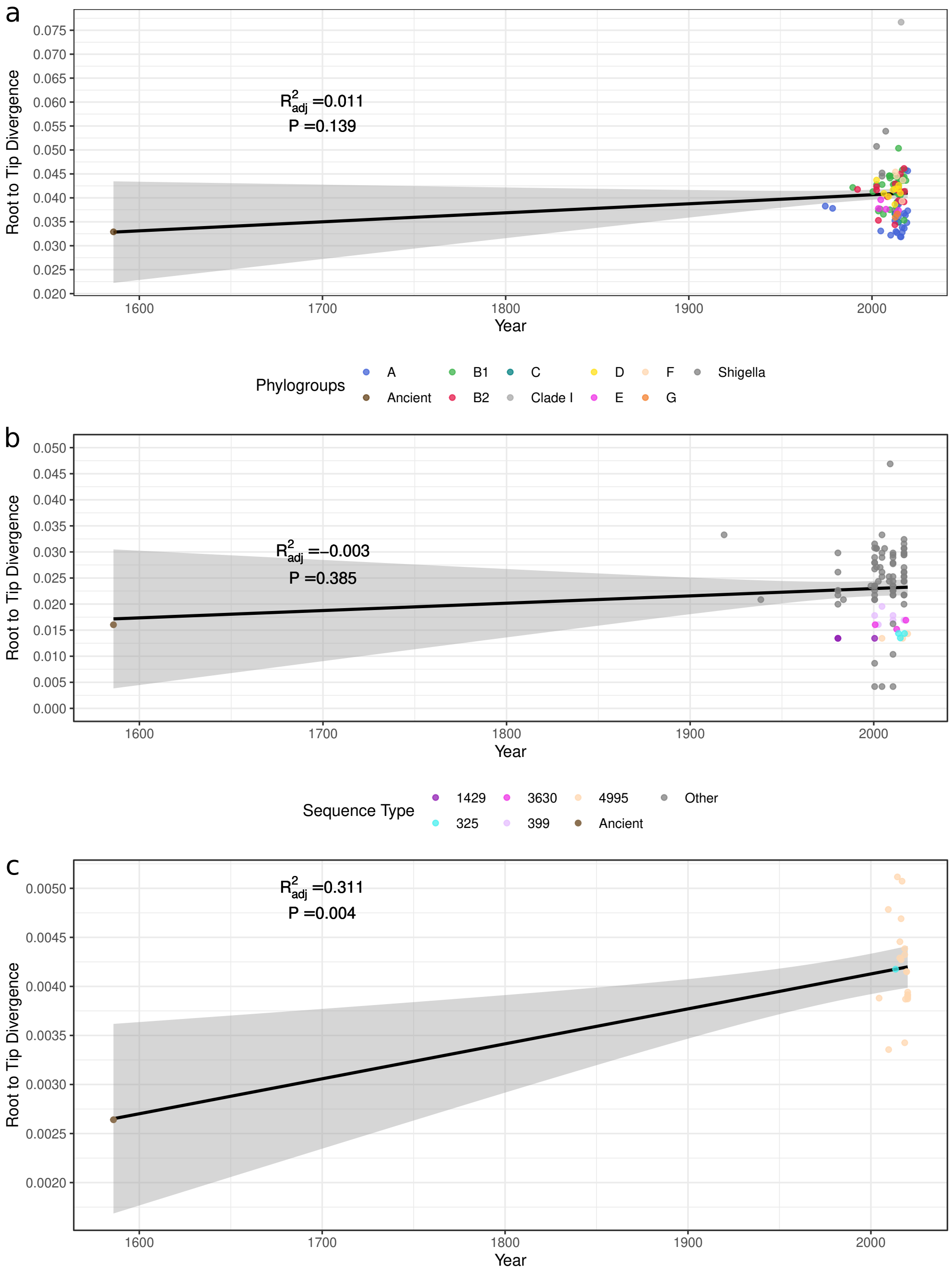

Supplemental Figure 8: **Identifying a Temporal Signal in the Phylogenies. a)** Root-to-tip divergence plot of the broad ML SNP phylogeny from Fig 3A. **b)** Root-to-tip divergence plot of the A0 ML SNP phylogeny from Fig 3B. **c)** Root-to-tip divergence of the ST4995 ML SNP phylogeny from Fig 3C. `TEMPest` was used to calculate the divergences and the trees were rooted using the heuristic mean squared method. A linear regression was used to determine the significance of the divergence. The shaded regions represent the 95% confidence interval.

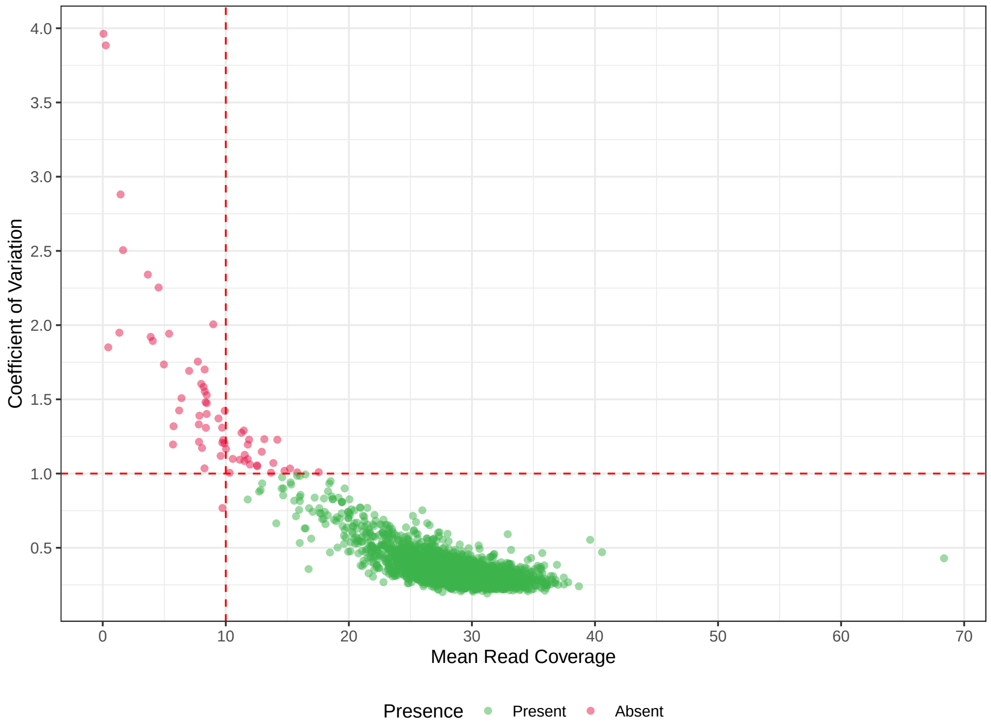

Supplemental Figure 9: **Scatter plot of the core genome coverage in our ancient genome.** The thresholds for gene presence – minimum 10× mean gene coverage and a CV ≤ 1 – are illustrated by the dashed red lines.

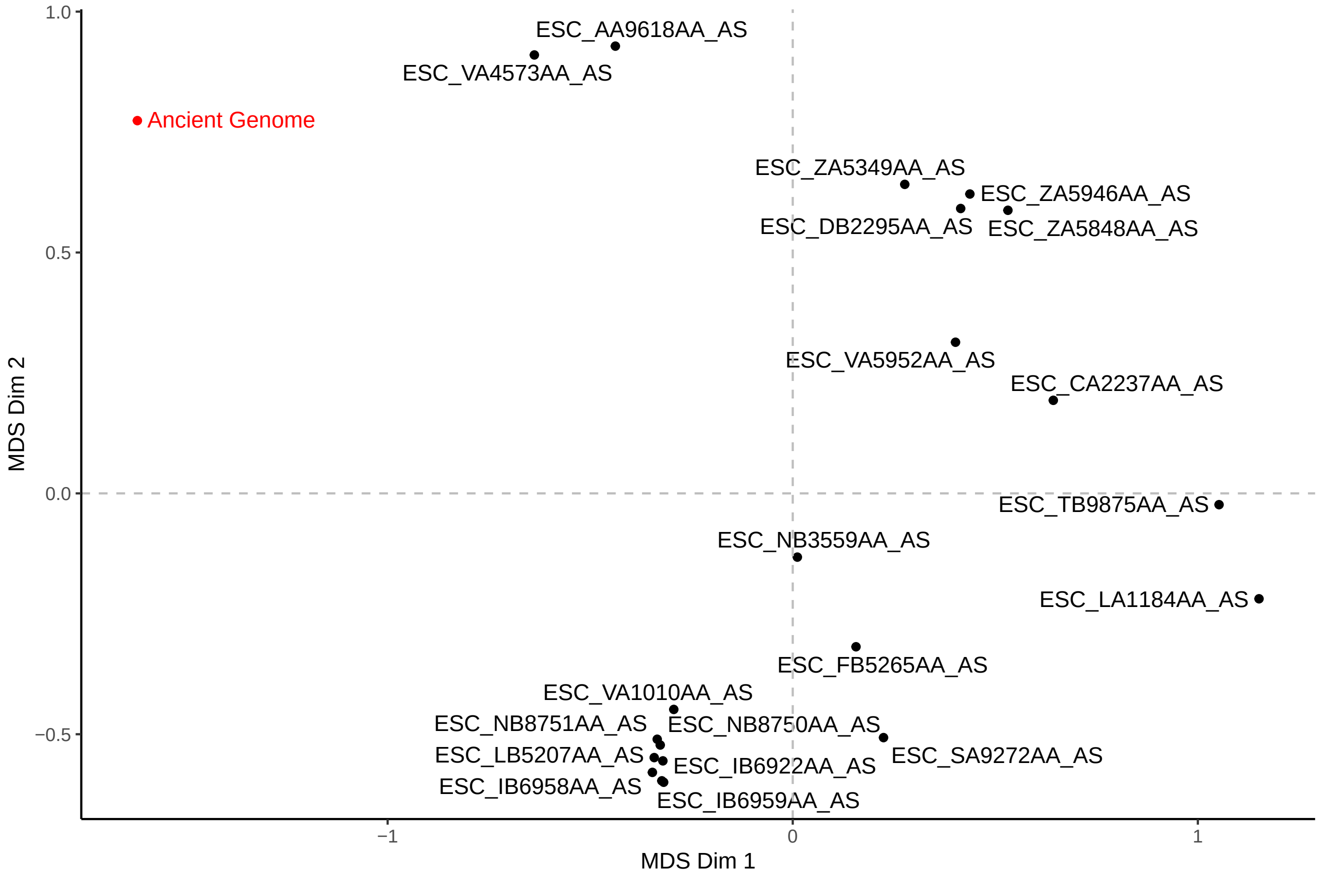

Supplemental Figure 10: **Venn Diagram of Genes found in ST4995**. Genes were identified with the A0 pangenome. For FSIS11816402 and ATCC11229 `Prokka` and `Roary` determined if a gene was present. In the ancient genome, a gene was considered present if it had an average sequence depth of at least 10× and a CV ≤ 1.

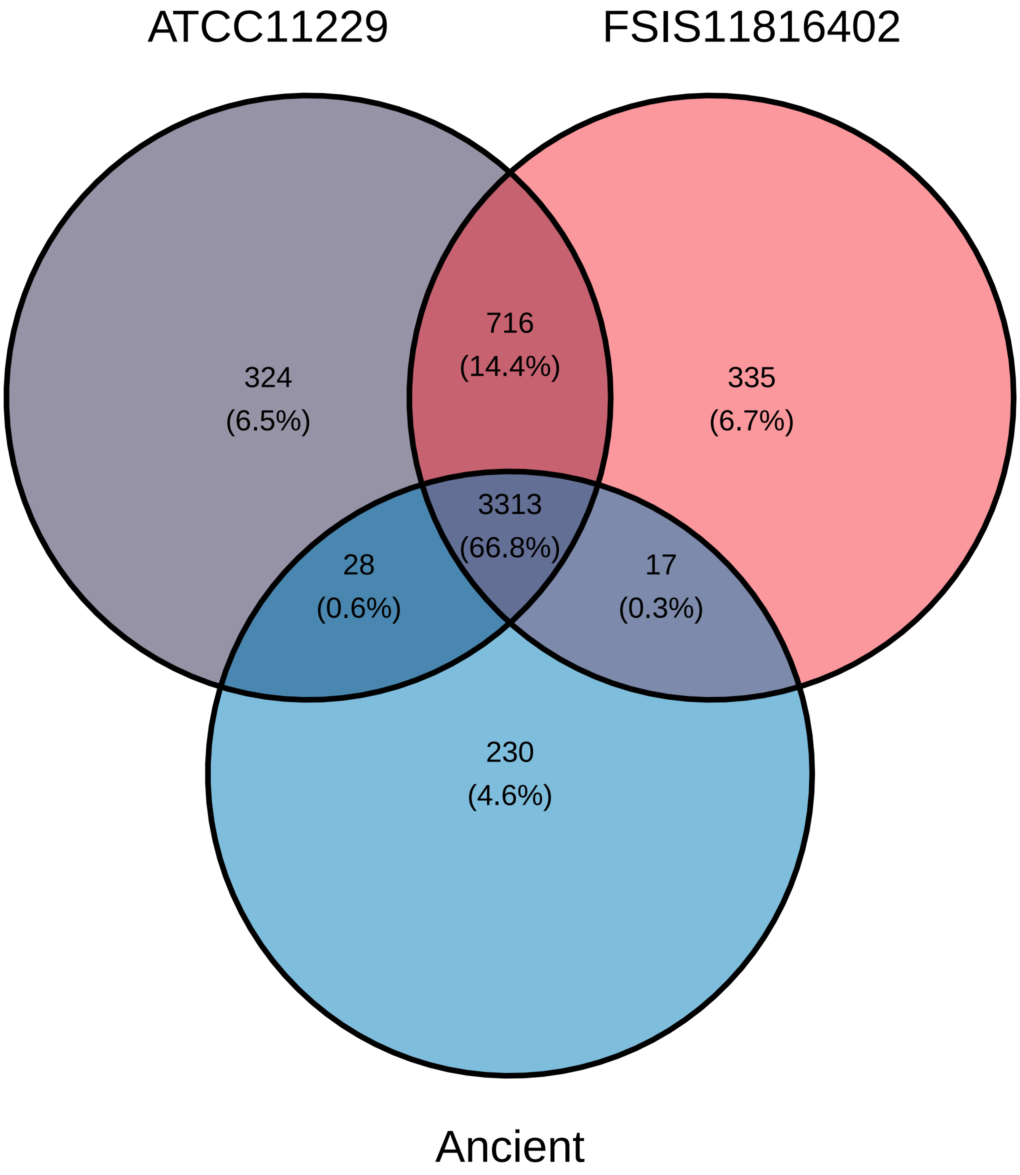

Supplemental Figure 11: **PCoA of accessory gene P/A in ST4995**. Genes were identified using a ST4995 pan-genome. `Prokka` and `Roary` were used to determine if a gene was present in the modern strains. For the ancient genome, a gene was considered present if it had an average sequence depth of at least 10$\times$ covering and a CV ≤ 1.

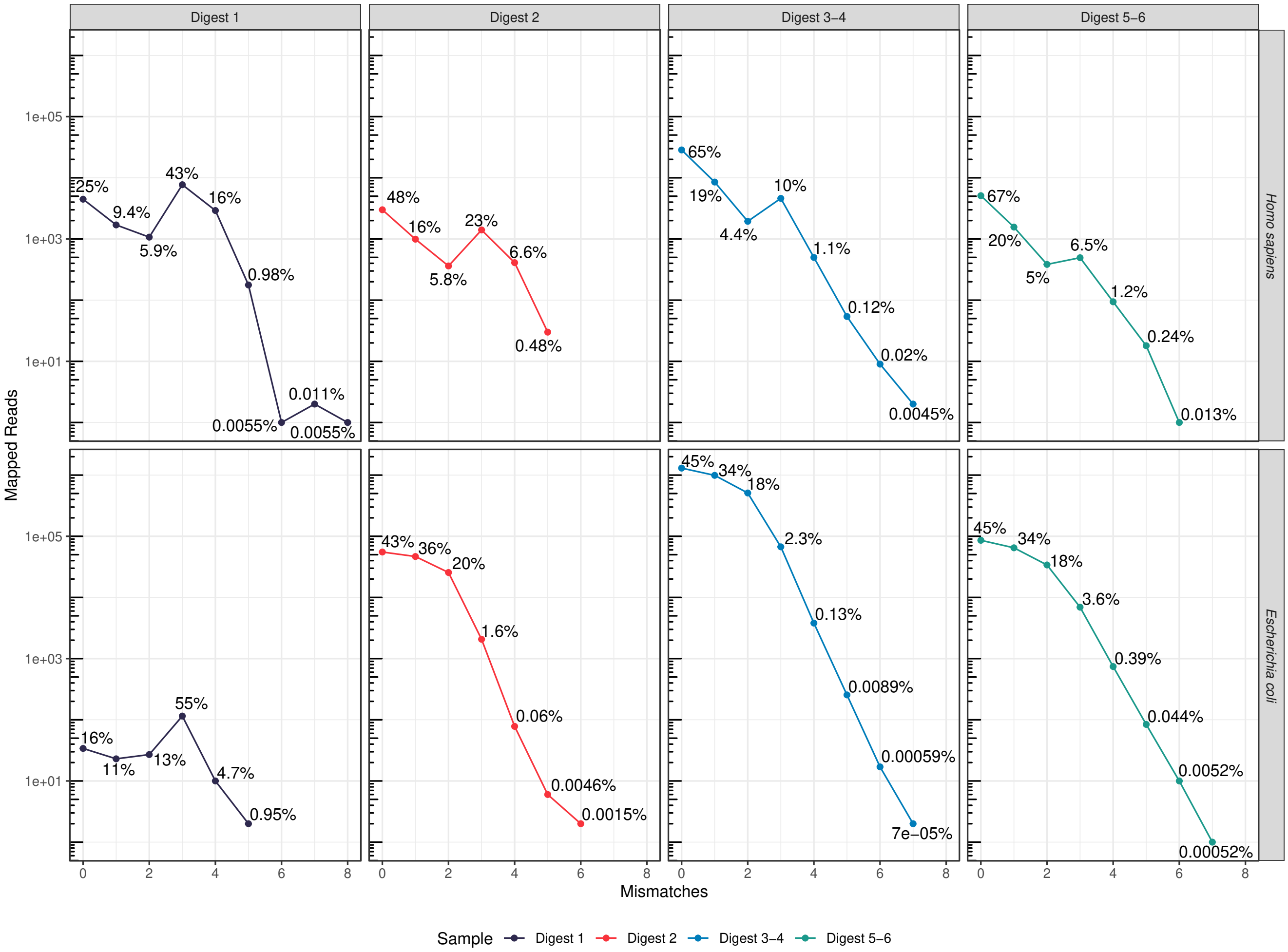

Supplemental Figure 12: **Edit distances of mapped fragments.** Proportion of *H. sapiens* and *E. coli* reads with a particular edit distance is shown. Edit distances were obtained using `samtools`.

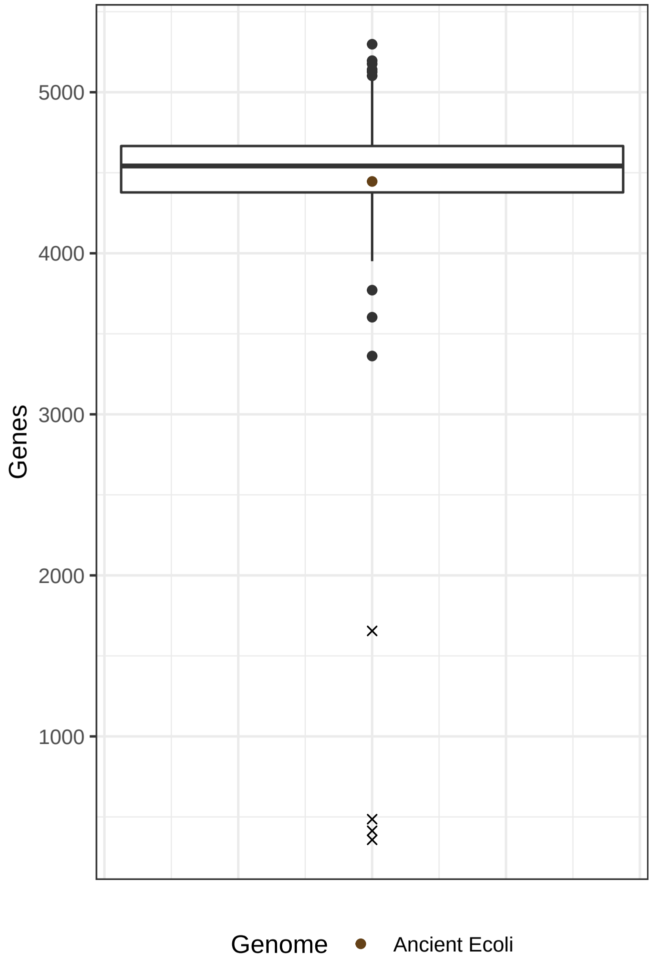

Supplemental Figure 13: **Box Plot of gene counts for *E. coli* genomes.** The counts for the ancient genome were added after the box plot was made to ensure visibility and prevent any biasing. Crosses are outliers that were subsequently removed from the rest of presence/absence analysis. n = 451 *E. coli* genomes. The box shows the first and third quartile, with the median indicated by the solid line. The whiskers represent 1.5× interquartile range at 3946 and 5098 genes for the lower and higher ranges respectively.

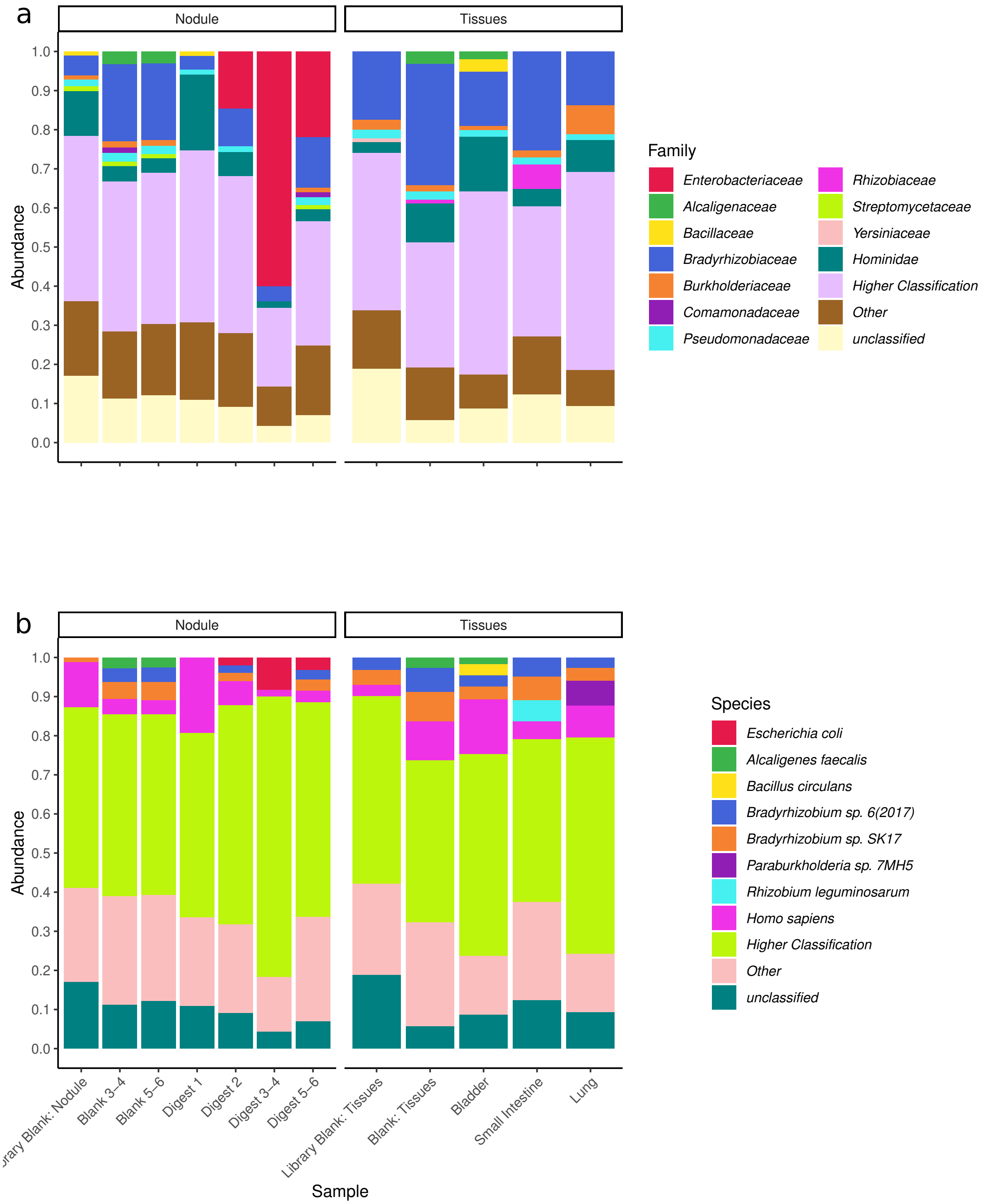

Supplemental Figure 14: **Proportional taxon abundances of NASD1 samples per** `Kraken2`. **a)** represents counts at the family level while **b)** is for the species level. A 1% – 1.2% when plotting the family results – abundance filter was used to filter out low-abundance taxa. The numbers at the top of each sample indicates the count of reads which were classified at either taxonomic level.
