# Supplementary material for: A 16th century Escherichia coli draft genome associated with an opportunistic bile infection
Source: Commun Biol. 2022 Jun 16;5:599. doi: 10.1038/s42003-022-03527-1 (PMC9203756; doi:10.1038/s42003-022-03527-1)
Supplement: Supplementary file 3 — Description of Additional Supplementary Files [file 42003_2022_3527_MOESM3_ESM.pdf]

Description of Additional Supplementary Files

File name: Supplementary Data 1
Description: List of E. coli strains used to create the pangenomes and phylogenies File name:

File name: Supplementary Data 2
Description: Plasmid gene coverage and comparison of the ancient strain to FSIS11816402
